# A Review of the Quality of Physical Health Care Provided to Adult Patients Admitted to Mental Health Inpatient Settings Across East and Central North Wales in Line With the National Confidential Enquiry Into Patient Outcome and Death (NCEPOD) Recommendations

**DOI:** 10.1192/bjo.2024.535

**Published:** 2024-08-01

**Authors:** Zeenish Azhar, Saima Saima, Premraj Muthuvelu

**Affiliations:** Betsi Cadwaladr University Health Board, North Wales, United Kingdom

## Abstract

**Aims:**

1. Measure compliance with NCEPOD Recommendations in the quality of physical healthcare provided to adult patients admitted to a mental health inpatient setting across the East and Central areas of North Wales.
2. Guide further service development and improvement in the quality of physical health care provided in mental health inpatient settings in North Wales.

**Methods:**

1. A retrospective case notes audit of 10 patients each who were inpatient for at least one week duration on the adult mental health wards was conducted in April 2023 across the East and Central areas of North Wales.
2. The audit was conducted using the NCEPOD audit Toolkit for “Physical Health in Mental hospitals”.

**Results:**

1. Inpatients percentage (%) compliance against NCEPOD recommendation 1, 5, 6, 7, 9 and 11 was 0% for both East and Central areas of North Wales respectively.
2. Recommendation 2 had 65% compliance for Central vs 61% for East.
3. Recommendation 3 had 62% compliance for Central vs 25% for East.
4. Recommendation 4 had 88% compliance for Central vs 40% for East.
5. Recommendation 8 had 3% compliance for Central vs 20% for East.
6. Recommendation 10 had 100% compliance for Central vs 94 % for East.
7. Recommendation 12 had 72% compliance for Central vs 71% for East.

**Conclusion:**

1. Improve compliance with the NCEPOD recommendations in the quality of physical healthcare provided to adult patients in mental health inpatient settings.
2. Develop a Trust wide policy document for physical health care in mental health inpatient settings in North Wales as per NCEPOD recommendations.
3. Develop a new physical health assessment booklet for Betsi Cadwaladr University Health Board Mental Health and Learning Disabilities Division to be used by all inpatient staff for the provision of physical healthcare of mental health inpatients in line with the NCEPOD recommendations.

